# Low circulating chemerin levels correlate with hepatic dysfunction and increased mortality in decompensated liver cirrhosis

**DOI:** 10.1038/s41598-018-27543-6

**Published:** 2018-06-18

**Authors:** Paul Horn, Christian von Loeffelholz, Franziska Forkert, Sven Stengel, Philipp Reuken, René Aschenbach, Andreas Stallmach, Tony Bruns

**Affiliations:** 1 Department of Internal Medicine IV, Gastroenterology, Hepatology and Infectious Diseases, Jena University Hospital, Jena, Germany; 2 Integrated Research and Treatment Center, Center for Sepsis Control and Care (CSCC), Jena University Hospital, Jena, Germany; 3 Department of Anesthesiology and Intensive Care Medicine, Jena University Hospital, Jena, Germany; 4 Institute for Diagnostic and Interventional Radiology, Jena University Hospital, Jena, Germany

## Abstract

Nutritional status, infections, inflammation and extrahepatic organ dysfunction are critical factors for the progression of chronic liver disease. Chemerin is an immune-metabolically and chemotactically active adipokine and we hypothesized that it is associated with disease severity and prognosis in patients with advanced decompensated cirrhosis. Therefore, we measured serum concentrations of chemerin in a prospectively characterized cohort of 80 patients with decompensated cirrhosis and ascites and assessed the associations with markers of disease severity and short-term outcome at 28 days. In a subset of patients (n = 40), ascitic fluid chemerin was determined. Advanced liver disease was associated with decreased serum but not ascitic chemerin levels. Serum chemerin correlated with markers of hepatic function (total bilirubin, albumin, INR) and inversely correlated with indicators of portal hypertension (platelet count, gastrointestinal bleeding) but not with extrahepatic organ failure and systemic inflammation. Patients presenting with acute-on-chronic liver failure or infection did not exhibit altered serum or ascitic fluid chemerin concentrations. However, serum chemerin levels below 87 ng/ml predicted an increased risk for mortality or liver transplantation within 28 days independently of MELD and infections. We conclude that low serum chemerin is an independent adverse prognostic factor in patients with advanced decompensated cirrhosis.

## Introduction

During the last years, adipokines emerged as multifunctional circulating mediators playing a role in different diseases such as obesity, diabetes mellitus, autoimmune diseases and chronic liver diseases including cirrhosis^1–7^. Chemerin was first described in 2003, when it could be isolated from malignant ascites and demonstrated chemotactic properties for antigen-presenting cells^8^. It is secreted from many tissues, predominantly adipose tissue and liver, as inactive prochemerin, which is proteolytically cleaved to several isoforms with different activity and spatiotemporal distribution^1,9,10^. Chemerin acts mainly via its receptor chemokine like receptor 1 (CMKLR1) on monocytes and parenchymal cells, but additional effects are mediated via G protein coupled receptor 1 (GPR1) and C-C chemokine receptor-like 2 (CCRL2). It has been described to modulate immune function by exerting both pro- and anti-inflammatory effects as well as antimicrobial properties^11,12^. Chemerin is associated with markers of the metabolic syndrome in clinical cohorts and is able to induce insulin resistance in mouse models, especially in skeletal muscle while acting as an insulin-sensitizing agent in adipocytes^2,13–15^.

Although chemerin correlates with necroinflammatory activity and fibrosis in non-alcoholic fatty liver disease (NAFLD) and in viral hepatitis it remains controversial whether chemerin contributes to the pathogenesis of liver disease^16–18^. Recent data suggest that hepatic chemerin gene expression is reduced in severe non-alcoholic steatohepatitis (NASH)^19^. In line with these results, negative associations have been described for chemerin with liver function and disease severity in patients with hepatocellular carcinoma (HCC) and cirrhosis^6,7^. These studies included patients with rather low-grade liver dysfunction and did not show any prognostic association of chemerin in liver cirrhosis.

Nutritional status, infections and inflammation as well as extrahepatic organ dysfunction are critical factors in the progression of chronic liver disease and complications of liver cirrhosis. Hepatic and extrahepatic organ failure as well as inflammation are implemented in current criteria for diagnosis of acute-on-chronic liver failure (ACLF) and sequential organ failure assessment in chronic liver failure (CLIF-SOFA) score^20^. Chemerin as a multifunctional adipokine might serve as a surrogate marker and mediator of different risk factors and complications but associations of chemerin with complications of cirrhosis and extrahepatic organ failure in acute-on-chronic liver failure have not been assessed yet.

Therefore, we determined serum concentrations of chemerin in a well-characterized cohort of 80 patients, who were hospitalized for decompensation of cirrhosis, to determine associations with hepatic and extrahepatic organ failure, bacterial infections, severity of inflammation and short-term prognosis in patients with decompensated liver cirrhosis and severe liver dysfunction. We focused on associations with overall prognosis, hepatic and extrahepatic organ failure, infections and inflammation as well as complications of liver cirrhosis.

## Results

### Patient characteristics

We included a total of 80 patients with decompensated cirrhosis and ascites. Patient characteristics are summarized in Table 1. Accounting for 76%, most patients were male. Median age was 57 years (range 24 to 83) and the majority presented with Child C cirrhosis with a median CPS of 10 points (range 7 to 14) and a median model for end-stage liver disease (MELD) score of 17 points (range 7 to 40). The predominant cause of cirrhosis was alcoholic liver disease (n = 63, 79%) and 57% of patients with alcoholic cirrhosis and 24% of patients without alcoholic aetiology reported alcohol consumption during last 30 days. Nine patients had suspected mild alcoholic hepatitis at study inclusion without the need for glucocorticoid therapy. In addition to decompensation with ascites, more than half of patients presented with bacterial infection and 12% with gastrointestinal bleeding as decompensating event.

**Table 1:** Patient characteristics.

	Study cohort (n = 80)
Male sex [n]	61 (76)
Age [years]	57 (50–64)
Aetiology of cirrhosis	
Alcoholic [n]	63 (79)
Viral [n]	5 (4)
NASH [n]	1 (1)
Others [n]	11 (14)
Active alcohol consumption yes/no/unknown	40/35/5
Alcoholic hepatitis [n]	9 (11.3)
Child-Pugh-Stage: A/B/C	0/29/51
Child-Pugh-Score	10 (9–12)
MELD	17 (11–23)
SOFA-CLIF	7 (4–9)
ACLF: 0/I°/II°/III°	56/14/5/5
Infections [n]	
SBP [n]	12 (15)
Infection other than SBP [n]	32 (40)
Upper gastrointestinal bleeding [n] (%)	10 (12.5)
Mean arterial pressure [mmHg]	87 (74–100)
Diabetes mellitus [n]	10 (12.5)
Insulin therapy [n]	6 (7.5)
Leukocyte count [/nl]	7.1 (5.0–11.7)
Thrombocyte count [/nl]	142 (96–189)
C-reactive protein [mg/l]	29.9 (13.3–60.9)
Creatinine [µmol/l]	85.5 (63.3–148.0)
Bilirubin [mg/dl]	2.08 (0.99–5.15)
Albumin [g/l]	24 (20–31)
INR [AU]	1.5 (1.2–1.9)
ALT [µmol/(l × s)]	0.56 (0.37–1.05)

Continuous data are given as median and interquartiles; nominal data [n] are given as n and percentage of total number study patients. Abbreviations: ACLF, acute-on-chronic liver failure; ALT, alanine aminotransferase; INR, international normalized ratio; MELD, model of end stage liver disease; NASH, non-alcoholic steatohepatitis; SBP, spontaneous bacterial peritonitis; SOFA-CLIF, sequential organ failure assessment in chronic liver failure.

### Chemerin in ACLF and acute infection

**Figure 1:**
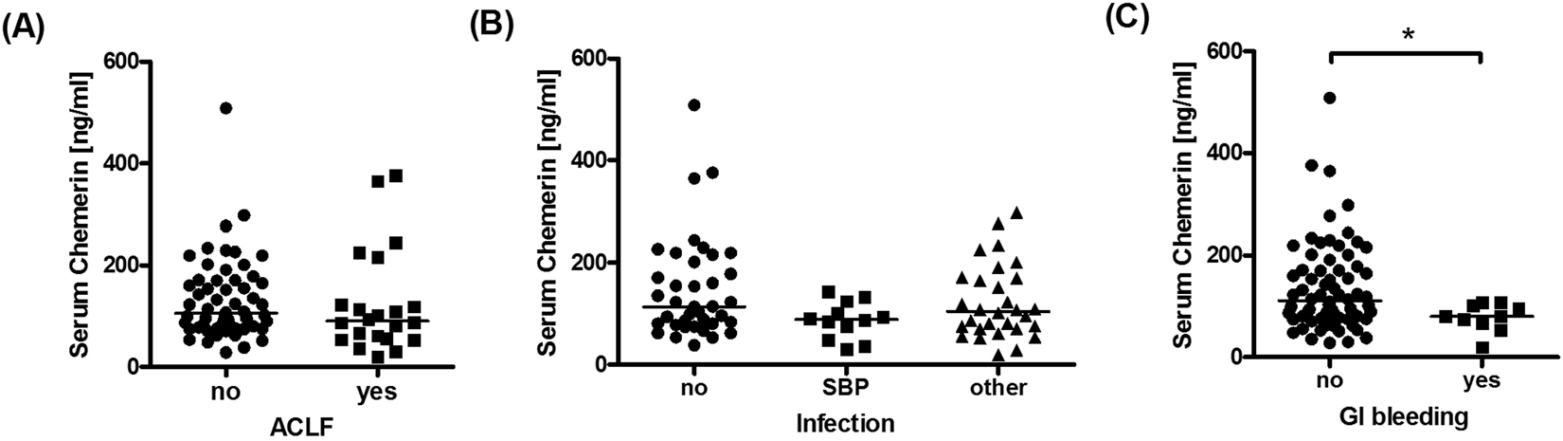
Chemerin concentrations in sera from patients with decompensated cirrhosis and ascites when stratified for the presence or absence of (**A**) acute-on-chronic liver failure, (**B**) bacterial infections, and (**C**) upper gastrointestinal haemorrhage as a decompensating event. Asterisk indicated p < 0.05 in Mann-Whitney U test or Kruskall-Wallis test with post-hoc Dunn’s test.

**Figure 2:**
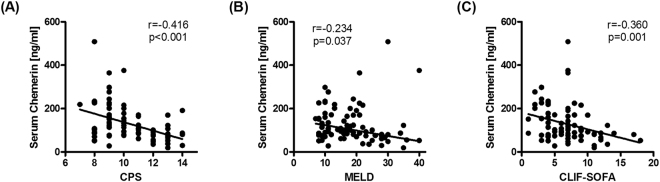
Correlation plots of serum chemerin concentrations with composite scores of disease severity. Non-parametric correlation of serum chemerin with (**A**) Child-Pugh-Score (CPS; r = −0.416, p < 0.001), (**B**) Model for End-stage Liver Disease (MELD; r_s_ = −0.234, p = 0.037) and (**C**) SOFA-CLIF (r_s_ = −0.360, p = 0.001) are shown.

Twenty-four patients presented with acute on chronic liver failure (ACLF) according to the EASL-CLIF criteria^20^. Twelve patients had spontaneous bacterial peritonitis (SBP) defined by an ascitic fluid neutrophil count greater than 250/µl in the absence of any intra-abdominal, surgically treatable source of infection^21^. Serum chemerin concentrations did not significantly differ between patients with and without ACLF (p = 0.265; Fig. 1A) and between patients without infection, SBP and patients with infections other than SBP (p = 0.165) (Fig. 1B). As chemerin concentrations have been described to be reduced in more advanced cirrhosis^6^, we determined the correlations of serum chemerin with classical composite scores of disease severity. In these analyses, chemerin negatively correlated with CPS (r_S_ = −0.416, p < 0.001), MELD (r_S_ = −0.234, p = 0.037) and SOFA-CLIF (r_S_ = −0.360, p = 0.001) (Fig. 2A–C).

As composite scores, CPS, MELD and SOFA-CLIF implement different clinical and clinic-chemical factors representing hepatic function and extrahepatic organ failure. Detailed analysis revealed that serum chemerin was negatively correlated with serum bilirubin levels (r_s_ = −0.451, p < 0.001), international normalized ratio (INR; r_s_ = −0.379, p = 0.001) and positively correlated with serum albumin (r_s_ = 0.289, p = 0.01) attributing to hepatic function, whereas no significant association was apparent with impaired kidney function as reflected by serum creatinine levels (r_s_ = 0.116, p = 0.307) (Table 2).

**Table 2:** Univariate correlation analyses with chemerin as dependent variable.

Independent variable	Spearman’s r	p-value
Bilirubin	−0.451	<0.001
INR	−0.379	0.001
Serum albumin	0.289	0.010
Serum protein	0.296	0.014
Serum creatinine	0.116	0.307
Thrombocyte count	0.330	0.003
SAAG	−0.155	0.179
Mean arterial pressure	0.276	0.013
C-reactive protein	−0.019	0.866
Leukocyte count	−0.166	0.142
Interleukin-6 (n = 79)	−0.179	0.113
Interleukin-10 (n = 72)	−0.196	0.098
TNF (n = 78)	0.062	0.589
Blood glucose (n = 77)	−0.127	0.270
BMI (n = 73)	−0.024	0.839
Visceral adipose tissue area (n = 44)	−0.157	0.309

Abbreviations: BMI, body mass index; INR, international normalized ratio; SAAG, serum-ascites albumin gradient; TNF, tumour necrosis factor (alpha).

While serum chemerin concentrations were not different between patients with or without esophagogastric varices (data not shown), patients who were hospitalized with gastrointestinal bleeding presented with significantly lower serum chemerin levels (p = 0.036; Fig. 1C). Additionally, we found a positive association of serum chemerin levels with platelet count (r_s_ = 0.990, p = 0.003) and mean arterial blood pressure (MAP, r_s_ = 0.276, p = 0.013) but not with serum ascites albumin gradient (SAAG, r_s_ = −0.155, p = 0.179) (Table 2).

As chemerin has been described to be associated with systemic inflammation and proinflammatory cytokines, we correlated serum chemerin with C-reactive protein (r_s_ = −0.019, p = 0.866) and total leukocyte count (r_s_ = −0.166, p = 0.142), which revealed no significant associations (Table 2)^12,22–24^. Consistent with these results, we also did not detect an association with the pro-inflammatory cytokines IL-6 and TNF (r_s_ = −0.179, p = 0.113 and r_s_ = 0.062, p = 0.589, respectively) or the anti-inflammatory cytokine IL-10 (r_s_ = −0.196, p = 0.098) (Table 2).

### Chemerin in ascitic fluid

Ascitic fluid chemerin concentrations correlated with serum chemerin (r_s_ = 0.627, p < 0.001; Suppl. Figure 1A) with lower chemerin concentrations in ascites than in serum (57.6 [28.2–99.5] vs. 101.9 [74.5–152.8] ng/ml, p = 0.001; Suppl. Figure 1B). In relation to peritoneal or serum albumin concentrations, the chemerin-to-albumin ratio was higher in ascitic fluid than in serum (5.8 × 10^−6^ [3.4–12.7] vs. 4.7 × 10^−6^ [3.0–6.1], p = 0.008, Suppl. Figure 1C). Notably, ascitic fluid chemerin did not differ between patients with and without SBP (p = 0.617; data not shown) and it did not correlate with total cell count or neutrophil count in ascites (r_s_ = 0.052, p = 0.754, n = 39 and r_s_ = −0.307, p = 0.106, n = 29, respectively; data not shown). Median serum-to-ascites ratio of chemerin was 1.92 [0.74–9.64], thus comparable to the predicted plasma-to-ascites ratio of 1.35–1.62^25^.

### Chemerin is inversely associated with skeletal muscle mass

**Figure 3:**
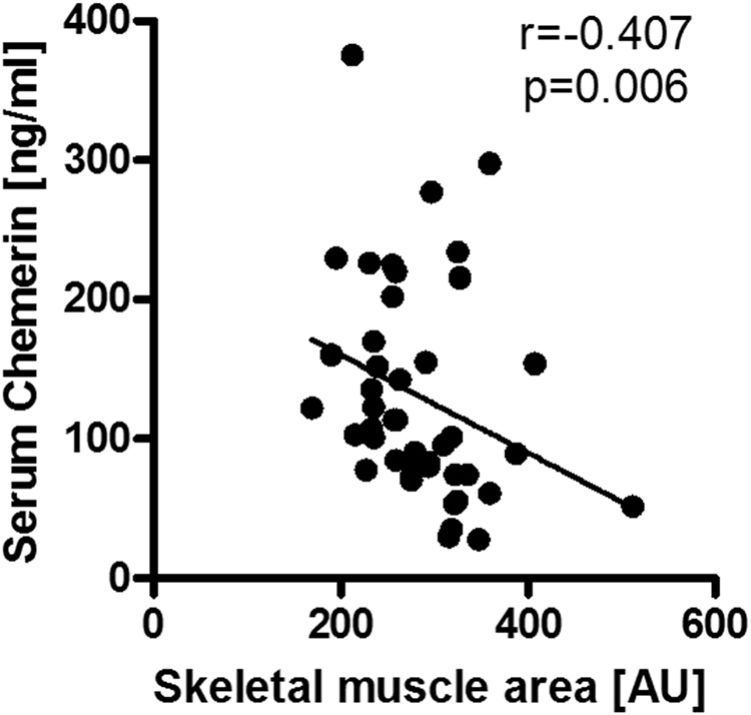
Correlation analysis of serum chemerin concentrations with skeletal muscle mass as determined by representative CT scans (r_s_ = −0.407, p = 0.006).

In previous studies in metabolic diseases, chemerin has been interpreted as a function of impaired glucose homeostasis, obesity or visceral adipose tissue mass^2,26,]^^27^. To address these confounders, we retrospectively extracted glucose levels and adjusted body mass index (BMI) from standard patient documentations and performed morphometric analyses of visceral adipose tissue and skeletal muscle area in available CT scans. We found no association with both glucose levels (r_s_ = −0.127, p = 0.27; n = 77) or adjusted BMI (r_s_ = −0.024, p = 0.839; n = 73). Although serum chemerin was not associated with visceral adipose tissue area (r_s_ = −0.157, p = 0.309; n = 44; data not shown), we observed a strong negative correlation with skeletal muscle area (r_s_ = −0.407, p = 0.006, n = 44; Fig. 3). Both visceral adipose tissue area and skeletal muscle area were not associated with prognosis or markers of hepatic function (data not shown).

### Low serum chemerin indicates poor short-term outcome

**Figure 4:**
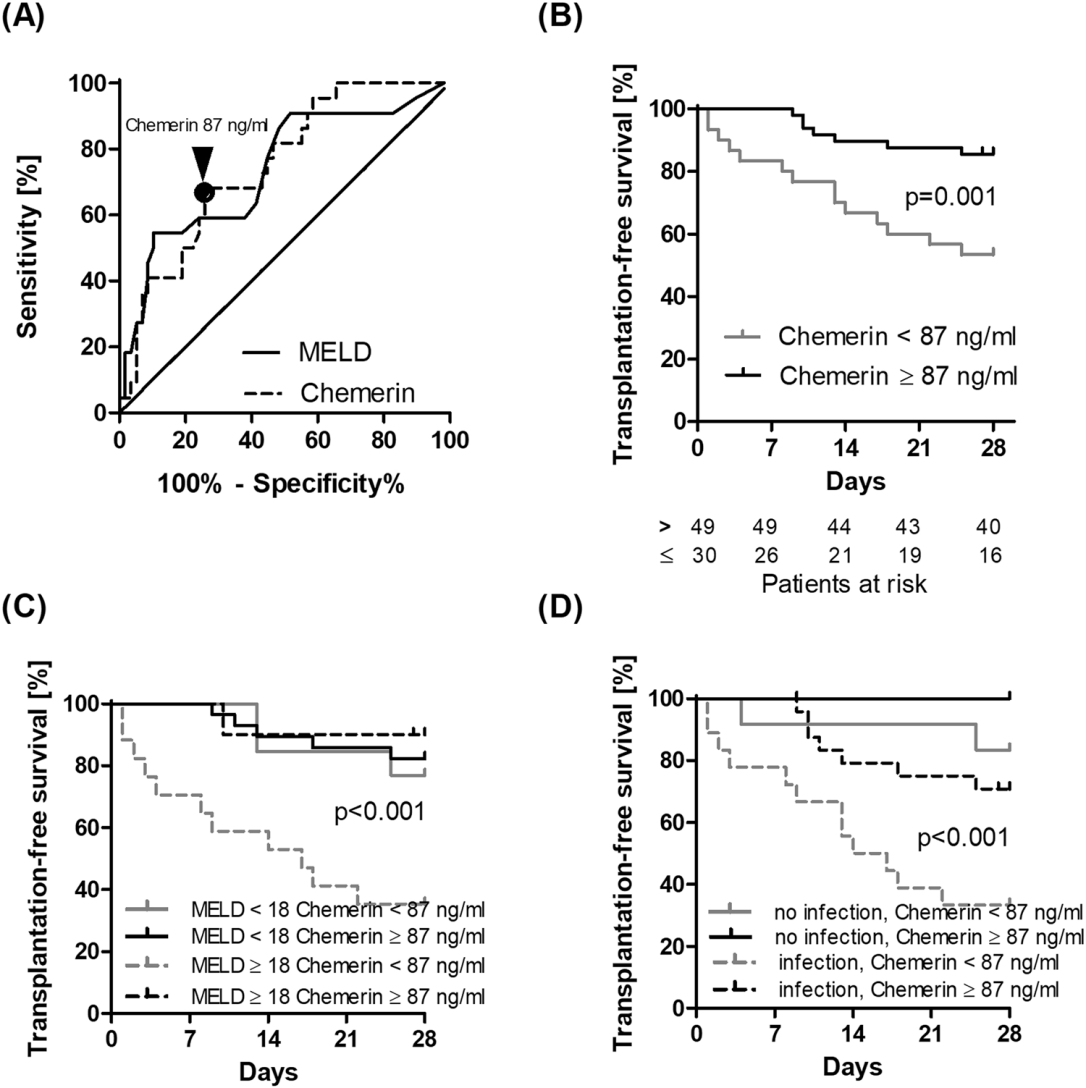
(**A**) ROC analysis for serum chemerin (dashed) and MELD (solid line) discriminating patients from patients who died or received transplantation within 28 days from patients who did not (AUROC 0.748 and 0.736, respectively). The optimum chemerin cut-off according to the Youden index is indicated. Kaplan-Meier analysis of 28 days transplant-free survival is shown (**B**) for patients stratified for serum chemerin concentrations (cut-off 87 ng/ml) alone and in combination with (**C**) dichotomized MELD score (overall p < 0.001) and (**D**) infection status (overall p < 0.001) is shown.

As low serum-chemerin was associated with hepatic dysfunction and more severe liver disease, we hypothesized that low chemerin levels indicate a risk of liver-related short-term mortality. Within 28 days after study inclusion, 17 (21%) patients died and five (6%) underwent liver transplantation. According to receiver operating characteristic (ROC) analysis, the diagnostic accuracy to predict transplant-free 28-day survival was 0.748 for serum chemerin and 0.736 for MELD (Fig. 4A). The Youden index identified an optimum chemerin cut-off of 87 ng/ml for further survival analyses. Circulating chemerin levels below 87 ng/ml were associated with highest risk for death and liver transplantation (OLT) compared to patients with higher chemerin serum levels (estimated cumulative transplant-free survival 53.3% [95% CI 34.2–69.1] versus 85.5% [95% CI 71.9–92.8]; p = 0.001; Fig. 4B).

Stratifying the study group revealed the highest risk for death or OLT in patients in the lowest tertile as compared to the median and highest tertiles (estimated cumulative OLT-free survival 51.9% [95% CI 32.0–68.6] versus 76.1% [95% CI 54.3–88.5] versus 92.3 [95% CI 72.6–98]; p = 0.003; Suppl. Figure 2). All but one liver transplantations were carried out because of high MELD (median score of 40). One patient received a transplant donation at a low MELD (score 21) due to rescue allocation but had high chemerin levels (107.38 ng/ml). When further stratified for MELD score or infections status, low chemerin was particularly predictive of poor transplant-free survival in patients at the highest risk with MELD of 18 or higher and/or infections (Fig. 4C,D). This association remained significant in time-to-death and in time-to-transplant analysis (Suppl. Figure 3).

Using Cox regression analysis, the univariate hazard ratio for mortality or liver transplant within the first 28 days was 4.28 [95% CI 1.74–10.51] (p = 0.002) in patients with serum chemerin <87 ng/ml. In multivariate Cox regression, low serum chemerin remained significantly associated with transplant or death within 28 days even after adjustment for MELD and infection (adjusted HR 2.93 [1.17–7.35], p = 0.022) (Table 3).

**Table 3:** Parameters at baseline associated with transplant-free mortality in patients with decompensated cirrhosis as assessed by Cox-regression analysis.

Variable	Univariate analysis		Multivariate analysis	
	Hazard ratio* [95% CI]	p-value	Hazard ratio* [95% CI]	p-value
Male Sex	1.025 [0.378–2.781]	0.961	—	—
Age (per 1-year increase)	1.008 [0.972–1.045]	0.663	—	—
MELD (per 1-point increase)	1.097 [1.049–1.148]	<0.001	1.090 [1.032–1.152]	0.002
Infection	9.708 [2.266–41.593]	0.002	6.895 [1.585–29.990]	0.010
Chemerin <87 ng/ml	4.277 [1.742–10.506]	0.002	2.934 [1.171–7.352]	0.022

*Hazard of mortality or liver transplantation within 28 days.

## Discussion

In this study, we provide a comprehensive exploratory evaluation of serum concentrations of the immunometabolically active adipokine chemerin as a promising biomarker in decompensated liver disease. We herein show that the lowest chemerin concentrations were found in patients with advanced cirrhosis as indicated by higher Child-Pugh, MELD and SOFA-CLIF scores. Notably, serum chemerin predicted short-term transplant-free mortality and correlated primarily with surrogates of impaired hepatic function and portal hypertension but not with extrahepatic organ failure, bacterial infection or the severity of systemic inflammation. Interestingly, the discriminative power of chemerin was best in the subgroup of patients with MELD scores of 18 or higher and/or infections, who have highest risk of mortality.

Previous data reported inconclusive results on the association of serum chemerin with liver function. In one cohort of patients with HCC, chemerin levels were associated with hepatic synthesis (prothrombin time, albumin), portal hypertension (low platelets) and hepatic inflammation (transaminases)^7^. A study in cirrhotic patients without HCC could confirm an association with coagulation (INR) but not with portal hypertension, bilirubin and hepatic inflammation^6^. Both studies included a lower number of patients with less advanced liver disease as compared to our study cohort^6,7^. In our study, chemerin levels below 87 ng/ml (normal value in men 95 ng/ml and in women 102 ng/ml, but varying due to lack of standardisation) indicated an increased risk for mortality and liver transplantation. Imai and colleagues used the median of observed chemerin levels as cut-off for survival analysis which may be a reason why they failed to show a prognostic association^7^. Our data confirmed associations of chemerin with liver function and implicate a prognostic value in patients with decompensated liver cirrhosis that seems to be limited to patients with severe disease indicated by MELD higher than 17. Notably, low chemerin levels indicated an increased short-term risk for liver transplantation and mortality, even after adjusting for MELD and infection. Given the number of 22 events, a multivariate Cox regression model including three variables must be interpreted with caution because of a risk of overfitting. Taken together, our findings should be verified in a larger prospective observational trial. Chemerin is processed to different isoforms with varying activity and spatiotemporal distribution^9^. Proportion of isoforms in decompensated cirrhosis should be studied and might add additional prognostic value, especially in patients with less severe disease.

It is tempting to speculate why chemerin serum concentrations are lower in patients with advanced cirrhosis. Chemerin is highly expressed in liver tissue and its concentration in central venous blood exceeds that in portal venous blood, pointing towards the liver as a main producer of circulating chemerin^4,10,22^. Conflicting data exists regarding the association of chemerin with inflammatory activity and fibrosis in NASH^3,28^, but in advanced stages NASH is associated with decreased hepatic chemerin expression and loss of functional parenchyma in severe liver cirrhosis might additionally contribute to reduced chemerin levels^19^. In addition, it has been shown that higher NAFLD activity is associated with reduced visceral adipose tissue chemerin gene expression in patients with NAFLD, pointing towards a role of adipose tissue in reduced chemerin levels in advanced liver disease^29^. No data exist regarding adipose tissue regulation of chemerin in portal hypertension or liver cirrhosis in general but it might be speculated that it is dysregulated due to altered blood flow, bacterial translocation and possibly subsequent adipose tissue inflammation. Despite an association of chemerin with activity of chronic liver disease in NAFLD, its role in pathogenesis remains unclear and chemerin has not been studied yet in pre-cirrhotic alcoholic liver disease. Additionally, to determine the role of liver functional reserves in regulation of chemerin, it might be of value to measure hepatic expression of chemerin in patients with cirrhosis and circulating chemerin in paired samples pre- and post-OLT. Although our results suggest a possible association with portal hypertension, decreasing portal pressure by portosystemic shunting did not affect circulating chemerin levels in a previous study and chemerin was not associated with ascites or varices in this study^6^. It has to be noted that most patients in our study were male and sex dimorphisms have been described for chemerin levels^30,31^. Chemerin levels and disease severity were not different between male and female patients in our study and sex was not associated with adverse outcome. Thus, unbalanced distribution of gender is unlikely to have influenced our results. Further, systemic inflammation and renal function are known modulators of circulating chemerin levels but did not show any association in our study cohort including patients with advanced decompensated liver disease^12,22,32^. As chemerin levels did not correlate with renal function, well established prognostic factors such as extrahepatic organ failure and hepatorenal syndrome should be considered when interpreting chemerin values as surrogates of survival.

Several limitations for a potential use of chemerin as a biomarker in liver cirrhosis must be noted. Most patients in our study cohort had alcoholic liver disease and our conclusions cannot be transferred to other aetiologies. The possible interference with extrahepatic organ failure, particularly renal impairment, remains challenging and due to the rather low number of study subjects we were not able to analyse whether the prognostic value of chemerin is restricted to certain prognostic outcomes. A larger prospective observational study is needed to adjust for these variables and solve this problem. Lack of standardisation of chemerin measurements with resulting high variance in reported values in healthy volunteers and different diseases additionally restricts the use of chemerin as a prognostic factor at the current state.

The immunomodulatory effects of chemerin are attributed to chemotactic properties on CMKLR1-positive cells^33^. While at the first glance this might implicate a proinflammatory role of chemerin in different diseases, the impact of chemerin on inflammation and infection is discussed controversially. We and others have previously shown an association of chemerin levels with systemic inflammation and sepsis in the absence of chronic liver disease^22,34^. In addition, it has been shown that chemerin exerts direct anti-microbial effects and can protect against lung inflammation in different animal models and against zymosan induced peritonitis^11,35–38^. Thus, very low chemerin levels might relate to impaired host defence in liver disease. As we did not observe an association with bacterial infection or inflammation, those are unlikely to act as confounding factors on the association of serum chemerin and outcome.

Although chemerin has first been described in ascites fluid from women with ovarian cancer^8^, to the best of our knowledge, this is the first report investigating chemerin concentration in portal-hypertensive ascites from patients with and without SBP. Although absolute chemerin concentrations were lower in ascitic fluid, the chemerin/albumin ratio was increased in ascites as compared to blood, indicating a peritoneal enrichment, albeit owing to local secretion or retention remains unknown. Although chemerin is concentrated in inflamed fluids and tissues, e.g. in osteoarthritis^39^, we did not observe increased chemerin levels in ascites in patients with SBP, so it seems unlikely that the enrichment of chemerin is associated with local inflammatory processes.

Chemerin has been identified as an adipokine and is associated with increased visceral adipose tissue mass, BMI and insulin resistance in type 2 diabetes^1,26,40,41^. In morbidly obese patients with NAFLD, chemerin levels have been correlated to visceral adiposity and BMI^28,42^. In contrast to the literature on patients without cirrhosis, we did not observe an association of chemerin levels with BMI, visceral adipose tissue mass or plasma glucose levels in this cohort of patients with cirrhosis and ascites. Though our observations are majorly limited by the retrospective extraction of BMI and glucose from standard patient documentation, they are in line with the report of Imai *et al*., where no association was found with BMI, HbA1c or HOMA-IR. Based on those data it can be speculated whether regulation of chemerin is distinct in decompensated cirrhosis compared to metabolic diseases. Therefore, the association of chemerin with metabolic parameters, including assessment of fasting glucose, insulin levels, homeostasis model assessment of insulin resistance (HOMA-IR) and lipid profiling should be assessed in future studies. According to a recent report, low leptin levels are associated with malnutrition and sarcopenia while other adipokines like adiponectin, resistin or ghrelin did not show any association, while chemerin was not studied^43^. Our results point towards a possible role of chemerin in sarcopenia in decompensated cirrhosis requiring confirmation by studies with prospective nutritional assessment. In contrast to our expectations, the prognostic associations of chemerin don’t seem to be related to reduced muscle mass as we observed higher chemerin levels in sarcopenia.

In summary, we herein demonstrate that low chemerin concentrations in patients with decompensated cirrhosis are a surrogate of hepatic dysfunction and portal hypertension and find no evidence for an association with extrahepatic organ failure, bacterial infection and systemic inflammation. Although circulating chemerin inversely correlates with MELD, in our study it provided additional prognostic information being an indicator of short-term risk for liver transplantation. Further studies are required to validate these findings in independent cohorts and unravel a mechanistic role of chemerin in the progression of advanced liver disease.

## Materials and Methods

### Study design and population

This study is a monocentric analysis of chemerin levels in a prospectively characterized cohort of patients with decompensated cirrhosis who were recruited to study the prognostic significance of bacterial DNA fragments^44^. Serum (n = 80) and ascitic fluid samples (n = 40) from patients who were hospitalized for decompensated cirrhosis with ascites between August 2010 and January 2013 in our centre were randomly selected for this study. The diagnosis of cirrhosis was made by histologic criteria or by a combination of clinical, laboratory and imaging data. Exclusion criteria were peritoneal carcinomatosis, secondary peritonitis, i.e. surgically treatable intra-abdominal source of infection including postoperative peritonitis after abdominal surgery, acute pancreatitis and tuberculous peritonitis. Child-Pugh-Score (CPS), Model of End stage Liver Disease (MELD) and sequential organ failure assessment in chronic liver failure (CLIF-SOFA) as composite scores of hepatic dysfunction and extrahepatic organ failure were calculated^20,45,46^. The diagnosis and grading of ACLF was made according to published criteria of the EASL-CLIF consortium^20^. Patients were followed-up for 28 days regarding the endpoints death and liver transplantation. All patients or their legal representatives gave written informed consent. The study was approved by the local ethics review board (Ethics committee of the Jena University Hospital, no. 2880-08/10 and 3683-02/03) and conformed to the ethical guidelines of the 1975 Declaration of Helsinki.

### Clinical chemistry

Laboratory data, except for chemerin and cytokine measurements, were prospectively collected as previously reported or carefully extracted from routine patient documentation. Laboratory tests were performed in certified Clinical Chemistry laboratories of Jena University Hospital^44^. Serum samples were to clot at room temperature over 30 min, and serum and ascitic fluid samples were centrifuged for 10 min at 1000 × g and stored at −80 °C until further analysis. Enzyme-linked immunoassays (ELISA) were used to assess the concentrations of chemerin, interleukin-6 (IL-6) and interleukin-10 (IL-10). Chemerin concentrations were measured using the commercially available Human Chemerin ELISA kit in duplicates (Biovendor, Germany). Serum IL-6 and IL-10 were determined using the human Interleukin-6 ELISA kit and ELISA human IL-10 assay (RayBiotech, Norcross, USA,). Serum Tumour Necrosis Factor alpha (TNF) was measured using the TNFα Human Matched Antibody Pair (ThermoFisher Scientific, Waltham, USA). Absorbance was determined in 96-well plates on a photo spectrometer (Infinite 200 Pro, TECAN, Männedorf, Switzerland) at 450 nm.

### Metabolic assessment

Plasma glucose levels, body height and weight were extracted from standard patient documentation, where available. For calculation of Body Mass Index (BMI), body weight was corrected according to the degree of ascites^43^. Body weight after paracentesis was used if available or subtracted with removed ascites volume. In patients without data for removed ascites volume, body weight was corrected according to clinical grade of ascites. In grade 1, grade 2 or grade 3 ascites we subtracted 5%, 10% or 15% of body weight, respectively.

Analyses of available native abdominal computer tomographic (CT) scans were performed by using dedicated workstation (AW Volume Share 5, General Electrics, Milwaukee, USA). On level with lumbar vertebral body 3, a set of ten slices with 5 mm thickness was obtained. As the first step, subcutaneous fat tissue was removed manually using the cutting function. After removal of subcutaneous fat, intra-abdominal muscles and vertebral body were circumscribed with the drawing function to exclude these tissues from measurement. Thereafter, visceral adipose tissue volume and paravertebral skeletal muscle volume were visualized by using following threshold parameters for Hounsfield-Units (HU): −190 to −30 HU for displaying visceral adipose tissue and −39 to 150 HU for displaying skeletal muscle tissue. By conversion of the created region-of-interest (ROI) into a volume, the total mass of adipose tissue and muscle tissue were calculated.

### Statistical Analyses

Statistical analyses were performed with SPSS 22.0 (SPSS Inc, USA) and Prism 5 (La Jolla, CA, USA). Data are given as median and interquartile range, if not stated otherwise. For comparisons between groups we used non-parametric Mann-Whitney U test, when comparing more than two groups, we performed Kruskal-Wallis-test with post-hoc Dunn’s adjustment. Wilcoxon test was used for paired samples. To test for correlation, Spearman’s rank coefficient was applied.

Time to event analyses were performed using univariate and multivariate Cox proportional hazards models correcting for co-factors. Patients who did not experience an end point (death or liver transplantation) within 28 days were right-censored after 28 days or at the time of last contact if lost to follow-up. Hazard ratios (HR) and Kaplan–Meier survivorship estimates were obtained. Discriminative abilities of continuous variables and ordinal scores to predict 28-day transplant-free mortality were assessed using the area under the receiver operating characteristic (ROC) curve. A p-value of <0.05 in a two-sided test was considered statistically significant.

## Electronic supplementary material

Supplementary Dataset 1
