# Supplementary material for: Low circulating chemerin levels correlate with hepatic dysfunction and increased mortality in decompensated liver cirrhosis
Source: Sci Rep. 2018 Jun 18;8:9242. doi: 10.1038/s41598-018-27543-6 (PMC6006249; doi:10.1038/s41598-018-27543-6)
Supplement: Supplementary file 1 — Supplementary Dataset 1 [file 41598_2018_27543_MOESM1_ESM.docx]

**Low circulating chemerin levels correlate with hepatic dysfunction and increased mortality in decompensated liver cirrhosis**

**Paul Horn*^1,2^; Christian von Loeffelholz^1,3^; Franziska Forkert^2^; Sven Stengel^2^; Philipp Reuken²; René Aschenbach^4^, Andreas Stallmach^1,2^; Tony Bruns^1,2^**

^1^Integrated Research and Treatment Center, Center for Sepsis Control and Care (CSCC), Jena University Hospital, Jena, Germany

^2^Department of Internal Medicine IV, Gastroenterology, Hepatology and Infectious Diseases, Jena University Hospital, Jena, Germany

^3^Department of Anesthesiology and Intensive Care Medicine, Jena University Hospital, Jena, Germany

^4^Institute for Diagnostic and Interventional Radiology, Jena University Hospital, Jena, Germany

**Supplementary Information**

**Supplementary Figures**

**Figure Legends**

**Supplementary Figure 1**

Analysis of chemerin concentration in ascitic fluid showing a correlation with serum chemerin (r=0.627. p<0.001) (**A**), a lower concentration as compared to serum concentrations (p<0.001) (**B**) and a higher chemerin/albumin ratio in ascitic fluid as compared to serum (p=0.008) (**C**). Chemerin levels in ascitic fluid were not different in patients with SBP or other infections compared to patients without infection (p>0.05) (**D**). Asterisk indicate p<0.05 in paired Wilcoxon test.

**Supplementary Figure 2**

Kaplan-Meier analysis of 28-days transplant-free survival for patients stratified for serum chemerin concentration tertiles (overall p=0.003 in log-rank Test).

**Supplementary Figure 3**

Kaplan-Meier analysis of overall survival (right censored at transplantation) (**A**), and time free from liver transplantation (**B**) for patients stratified for serum chemerin concentration (<87 ng/ml *versus* ≥87 ng/ml) and MELD score (<18 *versus* ≥18) (overall p<0.001, p=0.016 and p<0.001, respectively in log-rank test) and overall survival (**C**) and time free from liver transplantation (**D**) for patients stratified for status of infection (no infection *versus* infection) and serum chemerin concentration (<87 ng/ml *versus* ≥87 ng/ml) (overall p<0.001, p=0.001 and p<0.001, respectively in log-rank test).

**Supplementary Figure 1**

**
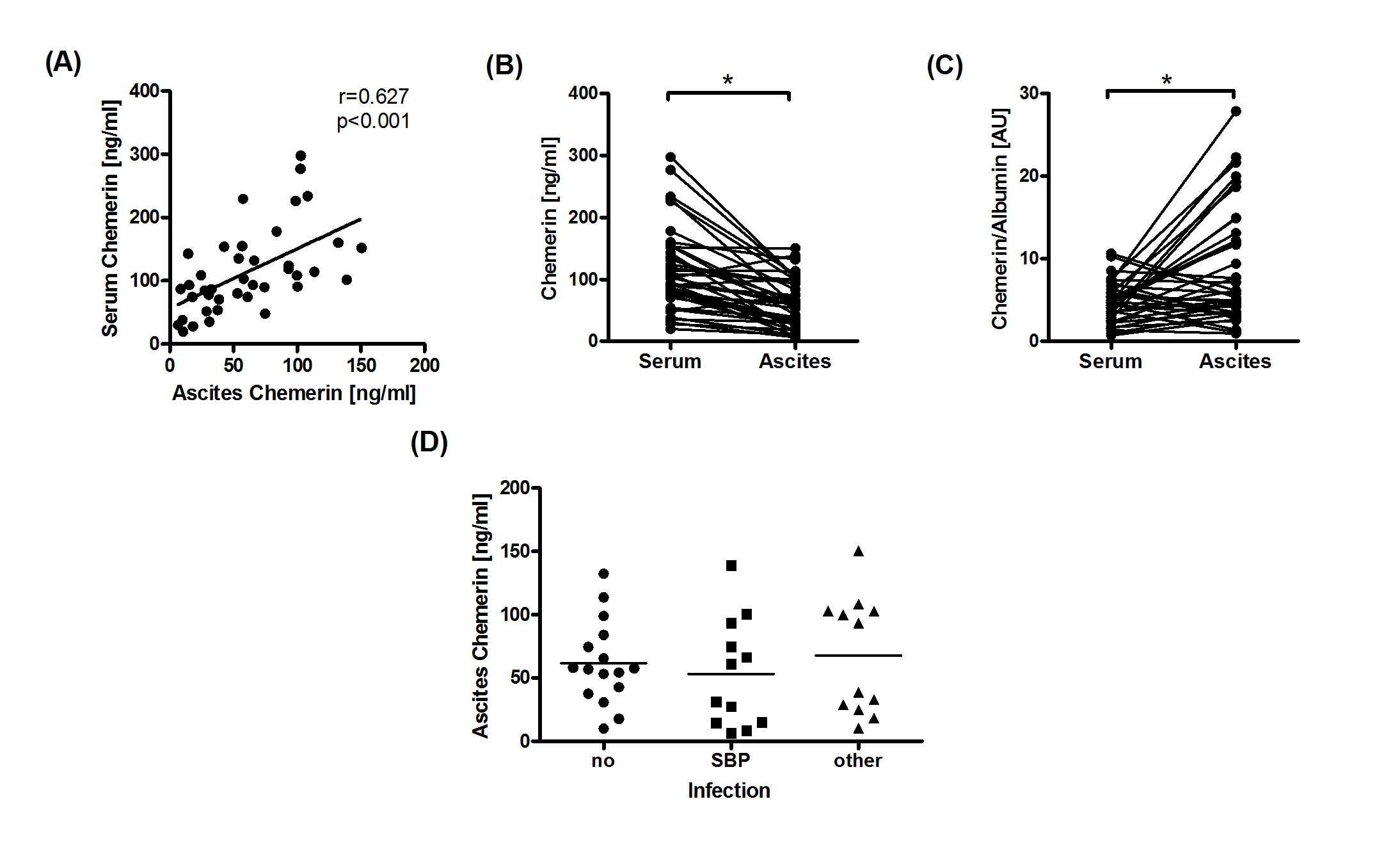
**

**Supplementary Figure 2**


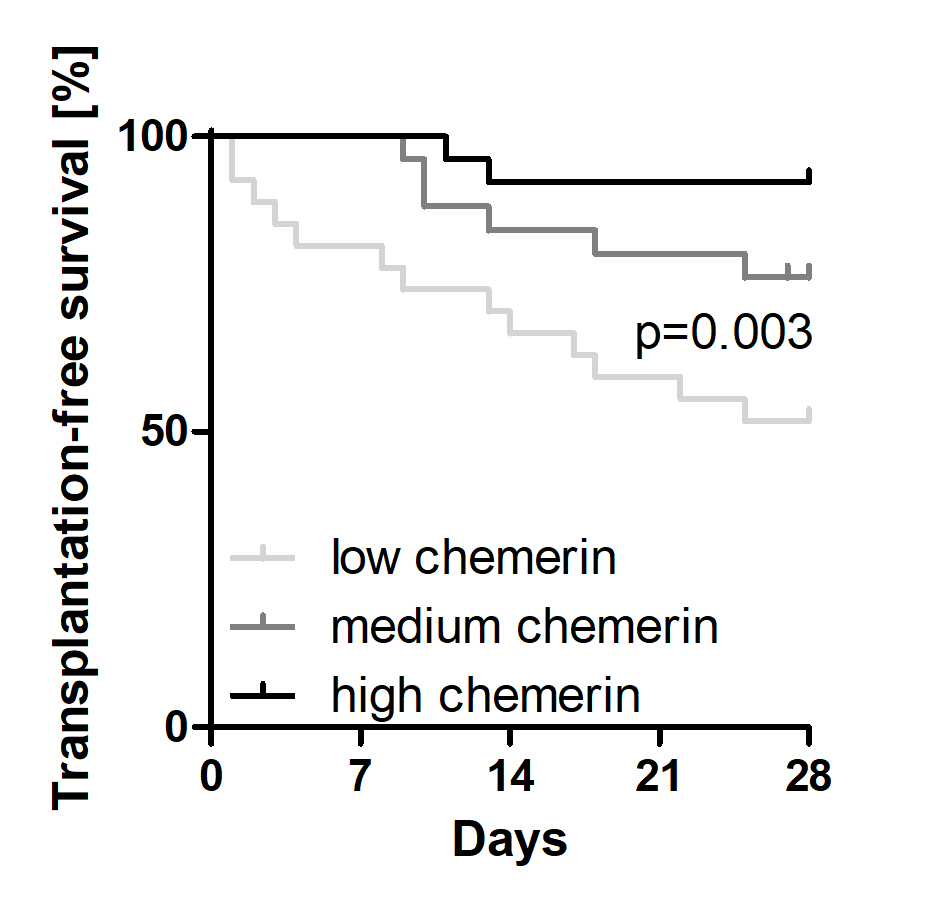


**Supplementary Figure 3
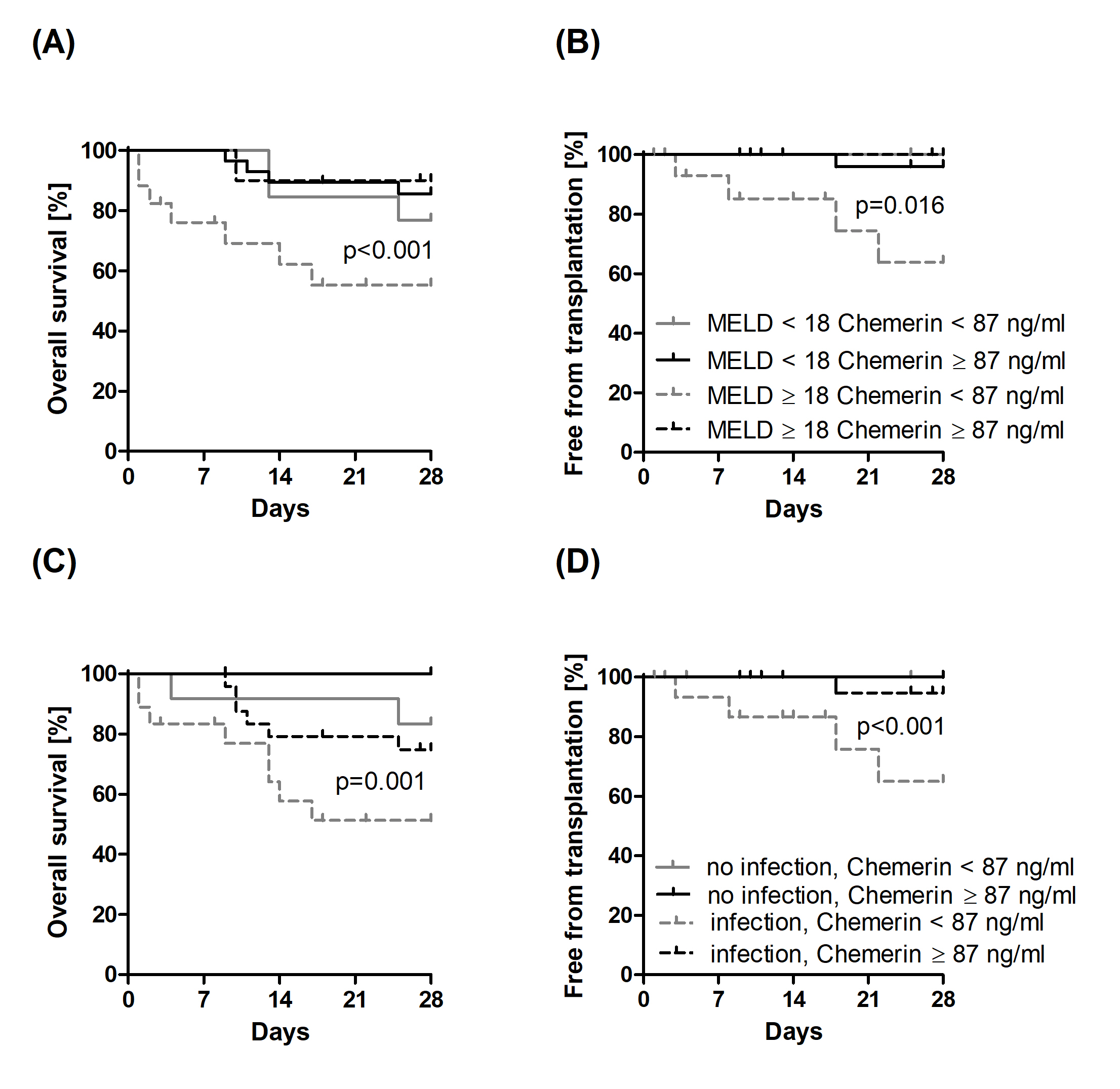
**
